# Taking stock and looking ahead: Behavioural science lessons for implementing the nonavalent human papillomavirus vaccine

**DOI:** 10.1016/j.ejca.2016.04.014

**Published:** 2016-07

**Authors:** Alice S. Forster, Jo Waller

**Affiliations:** Health Behaviour Research Centre, UCL, Gower Street, London, WC1E 6BT, UK

**Keywords:** Papillomavirus vaccines, Behavioural science, Patient acceptance of health care, Decision making, Psychological intervention

## Abstract

The development and licensing of a nonavalent human papillomavirus (HPV) vaccine has the potential to reduce morbidity and mortality from HPV-related cancers beyond that of first generation HPV vaccines. However, this benefit can only be realised if the offer of vaccination is accepted. Uptake of first generation HPV vaccines is not complete and shows huge global variation. In addition to practical and financial challenges to optimising coverage, behavioural issues explain a large proportion of the variance in vaccine receipt. This commentary draws on the findings of over a decade of behavioural science research seeking to understand uptake of first generation HPV vaccines, in order to anticipate challenges to implement the nonavalent HPV vaccine. Challenges include distrust of combination vaccines, uncertainty about long-term efficacy, distrust of a new and (perceived to be) untested vaccine, cost and uncertainty regarding interchanging doses of first generation and nonavalent vaccines and the appropriateness of revaccination. We use behavioural science theory and existing evaluations of interventions to increase uptake of vaccines to identify evidence-based approaches that can be implemented by vaccine stakeholders to address parents' concerns and maximise uptake of the nonavalent HPV vaccine.

## Highlights

• The benefits of human papillomavirus (HPV) vaccines can only be realised if uptake is optimal.
• Behavioural science can help anticipate challenges to uptake of new HPV vaccines.
• Challenges include distrust of combination vaccines and distrust of new vaccines.
• Cost and uncertainty about interchanging HPV vaccines may also affect uptake.
• Approaches to increase uptake of new HPV vaccines are identified.

## 1 Nonavalent human papillomavirus vaccines – a new advancement in prevention of human papillomavirus-related cancers

Human papillomavirus (HPV) is causally related to cancers of the cervix uteri, penis, vulva, vagina, anus and oropharynx. The development and worldwide implementation of vaccines against HPV have the potential to substantially reduce the burden of HPV-related cancers. First generation HPV vaccines (bivalent and quadrivalent HPV vaccines) offer protection against HPV types 16 and 18, which are known to cause up to 80% of HPV-related cancers (depending on cancer site) [1], [2]. The quadrivalent vaccine also offers protection against HPV types 6 and 11 which cause most anogenital warts. Vaccination is recommended to individuals aged 9 years and upwards, but works best if administered to HPV-naive individuals and in younger populations [3]. When the vaccines were first licensed, three doses were recommended but subsequent evidence suggests that two doses provide sufficient protection among younger girls [4].

More recently, a vaccine protecting against nine types of HPV has been licensed for use in the European Union (EU) and United States of America (USA, the nonavalent or nine-valent vaccine) [5]. This vaccine provides protection against five additional high-risk types which, together with HPV 16 and 18, cause around 90% of cervical cancers [6]. The vaccine may prevent an additional 4–18% of HPV-related cancers compared to bivalent/quadrivalent vaccines [7] and will provide equivalent protection against genital warts. A randomised controlled trial showed that the nonavalent vaccine was more effective at preventing HPV types 31, 33, 45, 52 and 58 than the quadrivalent vaccine and was non-inferior at preventing HPV types 6, 11, 16 and 18 [5]. The proportion of clinical adverse events was similar in the two groups, although adverse events related to the injection site (mild/moderate pain, swelling, redness, and itching) were more common in the group that received the nonavalent vaccine. Price permitting [8], it is likely that the nonavalent vaccine will be introduced into immunisation programs worldwide.

**Fig. 1:**
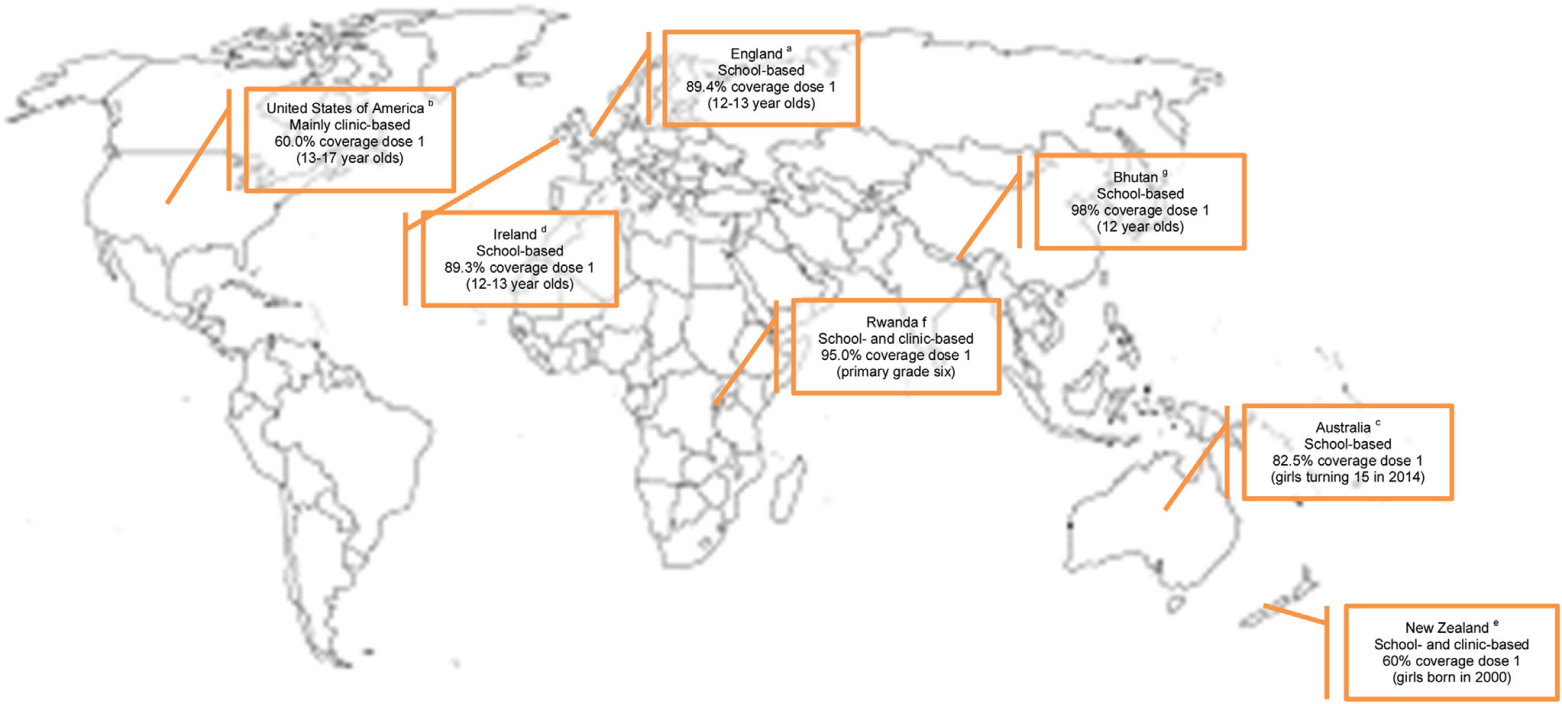
Female uptake and program delivery method of selected countries. ^a^https://www.gov.uk/government/uploads/system/uploads/attachment_data/file/487514/HPV_2014_15_ReportFinal181215_v1.1.pdf. ^b^http://www.cdc.gov/mmwr/preview/mmwrhtml/mm6429a3.htm#tab1. ^c^http://www.hpvregister.org.au/research/coverage-data/HPV-Vaccination-Coverage-2014. ^d^https://www.hpsc.ie/A-Z/VaccinePreventable/Vaccination/ImmunisationUptakeStatistics/HPVImmunisationUptakeStatistics/File,15198,en.pdf. ^e^http://www.health.govt.nz/our-work/preventative-health-wellness/immunisation/hpv-immunisation-programme. ^f^http://www.who.int/bulletin/volumes/90/8/11-097253/en/. ^g^http://www.sciencedirect.com/science/article/pii/S0264410X15007513.

While the development of vaccines against HPV represents a tremendous scientific advance, the promise of reduced incidence of HPV-related cancers can only be realised if the offer of vaccination is accepted. However, uptake is sub-optimal in most countries and shows wide global variation. With few exceptions [9], it tends to be highest in countries with school-based programs; for example, in Australia, uptake was 73% for girls turning 15 in 2014 [10]. By contrast, countries using clinic-based delivery often have lower uptake, exemplified by the USA where only around 40% of 13- to 17-year-old girls completed the series in 2014 [11] (see Fig. 1). It is striking that, with the exception of countries in South America, countries with organised HPV vaccination programs and good coverage tend to be those where the burden of disease is already low. In sub-Saharan Africa where the need is greatest, vaccination is generally not available, despite the on-going efforts of ‘Gavi, the Vaccine Alliance’. However, in this review, we focus on maximising uptake in countries where the vaccine is offered.

**Fig. 2:**
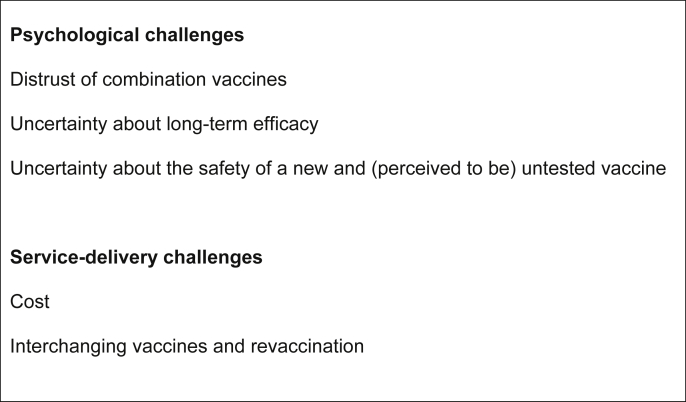
Challenges to maximise uptake of the nonavalent vaccine.

In addition to the practical and financial challenges to implementing vaccination programs and optimising coverage, behavioural issues have been shown to explain 40% of the variance in vaccine receipt [12]. Behavioural scientists have over a decade of experience conducting research into uptake of first generation HPV vaccines and can use the wealth of knowledge gained to anticipate challenges to implement nonavalent vaccines. Some challenges will be common to all vaccines (e.g. practical barriers to uptake) but others will be specific to HPV vaccination (e.g. low perceived risk of HPV infection) or to the nonavalent vaccine itself. In this commentary, we offer a behavioural science perspective on the challenges to maximise uptake of the nonavalent vaccine. Challenges are split into those that are psychological (the focus of behavioural science) and those related to service delivery, which are often raised by participants in behavioural science studies (Fig. 2).

## 2 What are the psychological challenges to vaccine uptake?

### 2.1 Distrust of combination vaccines – ‘Will the nonavalent vaccine overload the immune system?’

Studies have identified parental concern that vaccines can damage children by overloading their immune systems [13], as well as worry that particular ingredients in vaccines make them risky for their children [14]. Combination vaccines are considered particularly risky for both of these reasons [13], [14] as they are perceived to contain a greater number of ingredients mixed together, thus increasing the potency and potential for side-effects. The nonavalent vaccine is not a combination vaccine; however, parents are likely to be aware of first generation HPV vaccines and view the nonavalent vaccine as more complex (affording protection against nine as opposed to two or four HPV types).

### 2.2 Uncertainty about long-term efficacy – ‘I’ll wait to decide until there's more evidence that it will protect my daughter in her twenties’

Uncertainty about the duration of protection afforded by first generation HPV vaccines has been a prevalent concern, due to the relatively recent development of the vaccines, and the scientific uncertainty about the actual duration of protection [15] (although estimates suggest that protection will last decades [6]). For this reason, parents who do not expect their child to be sexually active in the near future may be particularly likely to delay HPV vaccination [16]. Unpublished data from our group (Forster, Rockliffe, Waller *et al.*) suggest that these parents believe that vaccination should occur close to sexual debut to maximise the duration of protection after potential exposure to HPV. Concerns about duration of protection are not limited to HPV vaccines [17]. The nonavalent vaccine may be seen by parents as going ‘back to square one’ in terms of the evidence of long-term efficacy, as it is newly developed.

### 2.3 Uncertainty about the safety of a new and (perceived to be) untested vaccine – ‘They don't know about the long-term side-effects’

One online focus group study of parents of vaccinated and unvaccinated girls, conducted in the USA, identified concern about side-effects of the nonavalent vaccine [18]. Worry about possible long-term side-effects of first generation HPV vaccines is a well-established barrier to vaccine acceptance, and centres on the lack of long-term surveillance data due to the vaccine's novelty [14], [15]. Parents who do not expect their child to be sexually active in the near future tend to feel they can afford to delay vaccination until more evidence about safety is available [19]. Parents may perceive that there is even less evidence on long-term safety of the nonavalent vaccine.

Concerns about vaccine safety are not unique to HPV vaccines. However, the impact of such concerns on vaccine coverage should not be underestimated. Pre- and post-licensing surveillance data show that HPV vaccines are safe [5], [20]. However, anecdotal reports of serious adverse events have been presented in the media, resulting in parents receiving conflicting information. Governments have had varying degrees of success in managing these reports. For example in the United Kingdom (UK), a girl died shortly after receiving the HPV vaccination, but her death was quickly attributed to a tumour in her chest [21]. Management of the event by public health authorities resulted in no noticeable impact on HPV vaccination coverage in the UK [22]. Conversely, uptake in Japan decreased significantly after the Ministry of Health asked local health authorities to stop promoting the vaccine following public reports of adverse events [23]. While public health officials might be able to contain vaccine concerns among their own population, vaccine sentiments travel globally.

## 3 What are the service-delivery challenges to vaccination uptake?

### 3.1 Cost – ‘I can't afford three doses of the vaccine’

Economic modelling suggests that nonavalent vaccination would be more cost-effective than quadrivalent if the additional cost per dose did not exceed $13 [8]. Even if the price of the nonavalent vaccine does not exceed this threshold, where payment for vaccination is not met by health services or medical insurance, cost is likely to prohibit vaccine receipt for some. This is particularly the case in developing countries, although ‘Gavi, the Vaccine Alliance’ has facilitated HPV vaccination for many individuals. Three doses of the nonavalent vaccine are required, compared to only two doses of first generation vaccines, which will increase cost for those paying for vaccination privately. Cost has previously been reported as a concern about first generation and nonavalent vaccines^1^ [18], [24] and may result in individuals starting but not completing the vaccine series.

### 3.2 Interchanging vaccines and revaccination – ‘Can my daughter have both a first generation vaccine and the nonavalent one?’

While the nonavalent vaccine is being introduced, parents whose children have had one or two doses of a first generation HPV vaccine may query whether it is possible to complete the series with the nonavalent one. In addition, parents whose children have completed the series with one of the first generation vaccines may see the benefits of the nonavalent vaccine and consider whether it is appropriate to revaccinate with the new vaccine. However, there have been no studies looking at the inter-changeability of HPV vaccines and there is only preliminary evidence that vaccination with the nonavalent vaccine is safe following series completion with the quadrivalent vaccine [25]. Temporary guidance on this issue for individuals exists [25] but clinical data are needed to inform vaccination programs.

## 4 What can be done to increase uptake of the vaccine?

Where parents are given the option to choose between first generation and nonavalent vaccines, uptake may not fall, as parents with concerns can choose the more established first generation alternative. However, where choice of HPV vaccine is restricted by health insurers/health authorities, the vaccine offered may affect uptake. In such circumstances, vaccination stakeholders would benefit from the insights that behavioural science can offer about interventions for maximising uptake.

**Fig. 3:**
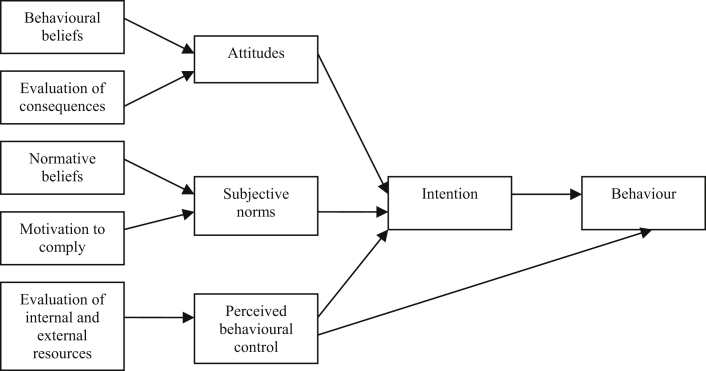
Theory of planned behaviour.

**Fig. 4:**
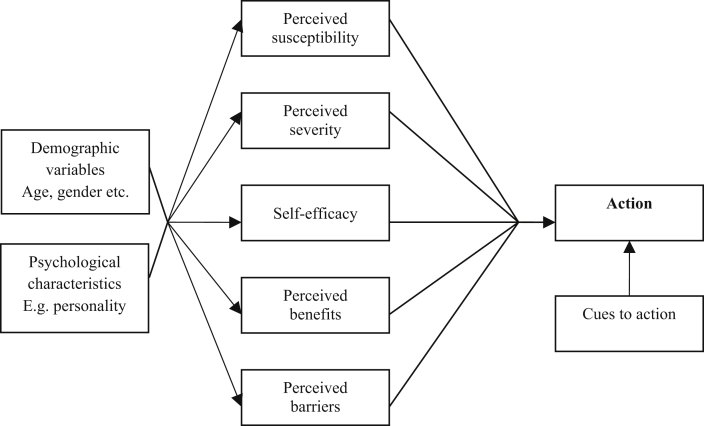
Health belief model.

The behavioural scientist's toolkit includes a number of theories that have informed research seeking to understand the psychosocial factors influencing HPV vaccine receipt, as well as to identify targets for interventions to increase uptake. Two key theories have been used often in this field: the theory of planned behaviour (TPB) [26] and the health belief model (HBM) [27] (Fig. 3, Fig. 4). The TPB suggests that behaviour is directly informed by behavioural intentions and an individual’s perceived behavioural control (PBC, whether they believe they have control over performing the behaviour). Behavioural intentions are influenced by a person's attitudes, subjective norms (beliefs about what others would want them to do and their motivation to comply with this), as well as their PBC. The HBM suggests that six constructs influence whether a behaviour will be performed: perceived susceptibility and severity of the illness being prevented, perceived benefits and barriers to engaging in the recommended preventive behaviour, self-efficacy (akin to PBC) and cues to action (triggers that prompt behaviour). Given that many of the challenges discussed relate to the perceived costs and benefits of the nonavalent vaccine (HBM) and individuals' attitudes towards it (TPB), vaccine stakeholders seeking to increase uptake of the nonavalent vaccine may wish to consider intervening to change constructs of the HBM and TPB as these theories have been shown as a whole to predict vaccination behaviour [12]. Additional motivators to vaccination may be considered as targets, for example, although smoking status is associated with HPV positivity, to our knowledge, interventions to increase uptake of HPV vaccination have not been directed specifically at individuals who smoke.

There is evidence that some behavioural interventions are effective at increasing uptake of HPV vaccination [28], [29] and these may be modified to address concerns about the nonavalent vaccine. Educational interventions aimed at both parents and adolescents have generally not demonstrated effectiveness at increasing HPV vaccination uptake [28] (although improvements in adolescents' attitudes towards vaccination are observed), whereas practice- and community-based interventions (such as reminders and school-based programs) are likely to be more successful [29]. We also know that the use of ‘presumptive’ communication (‘your child is due for the HPV vaccine’) is associated with greater vaccine acceptance compared with ‘participatory’ communication (‘what do you want to do about the HPV vaccine?’) [30]; although debates about which of these is most appropriate, given the need for informed consent, are ongoing. Interventions have also been developed to minimise girls' anxiety about having an injection, for delivery in school-based programs (to avoid mass syncope) [31]; however, there is not yet evidence of the efficacy of such interventions.

Vaccination stakeholders may also consider addressing some of the concerns that parents may have about the nonavalent vaccine. Parents' potential concerns about the safety and efficacy of the nonavalent vaccine may be clarified by explaining what testing has been done and why efficacy is likely to be sustained. Long-term studies of cohorts who received the quadrivalent vaccine have not shown any reduction in immunity, suggesting that the nonavalent vaccine may also provide long-term protection [5]. The nonavalent vaccine demonstrated a good safety profile and it has been licensed for use in the USA and EU. Parents' preferences to delay vaccination to maximise the time that their child is protected against HPV (because they are concerned about long-term efficacy and safety coupled with their belief that their child will not be sexually active soon) may be challenged by explaining that the vaccine leads to a better immune response if delivered when an individual is younger. Finally, where evidence is lacking regarding the inter-changeability of HPV vaccines and revaccination, health professionals will need to be sufficiently prepared to answer queries.

## 5 Conclusions

Nonavalent HPV vaccines represent a new opportunity to prevent HPV-related cancers. Drawing on the evidence-base generated by behavioural science with regard to first generation HPV vaccines, vaccination stakeholders can anticipate parents' concerns and be prepared to address them. Modifying interventions that we know are effective at improving HPV vaccination uptake, so that they tackle concerns specific to the nonavalent vaccine, may help to maximise uptake.

## Funding

This work was supported by Cancer Research UK (grant numbers C49896/A17429 to ASF, C7392/A17219 to JW). The funder played no role in the writing of this current perspective report and in the decision to submit the article for publication.

## Conflicts of interest statement

None declared.

## Footnotes

1 Note that these studies were conducted in countries where HPV vaccination is covered by national health authorities or medical insurance.

## References

[1] Forman D., de Martel C., Lacey C.J. (2012). Global burden of human papillomavirus and related diseases. Vaccine.

[2] National Institutes of Health NCI. HPV and cancer. [accessed 29.01.16]. http://www.cancer.gov/about-cancer/causes-prevention/risk/infectious-agents/hpv-fact-sheet.

[3] Block S.L., Nolan T., Sattler C. (2006). Comparison of the immunogenicity and reactogenicity of a prophylactic quadrivalent human papillomavirus (types 6, 11, 16, and 18) L1 virus-like particle vaccine in male and female adolescents and young adult women. Pediatrics.

[4] Dobson S.M., McNeil S., Dionne M. (2013). Immunogenicity of 2 doses of hpv vaccine in younger adolescents vs 3 doses in young women: a randomized clinical trial. JAMA.

[5] Joura E.A., Giuliano A.R., Iversen O.E. (2015). A 9-valent HPV vaccine against infection and intraepithelial neoplasia in women. N Engl J Med.

[6] Herrero R., González P., Markowitz L.E. (2015). Present status of human papillomavirus vaccine development and implementation. Lancet Oncol.

[7] Saraiya M., Unger E.R., Thompson T.D. (2015). US assessment of HPV types in cancers: implications for current and 9-valent HPV vaccines. J Natl Cancer Inst.

[8] Brisson M., Laprise J.F., Chesson H.W. (2016). Health and economic impact of switching from a 4-valent to a 9-valent HPV vaccination program in the United States. J Natl Cancer Inst.

[9] Hopkins T.G., Wood N. (2013). Female human papillomavirus (HPV) vaccination: global uptake and the impact of attitudes. Vaccine.

[10] National HPV Vaccination Program Register. National (Australia) HPV 3 dose vaccination coverage for females turning 15 years of age in 2014. [accessed 10.02.16]. http://www.hpvregister.org.au/site/DefaultSite/filesystem/documents/Coverage-Data/2015/National%203%20dose%20coverage%20for%2015%20year%20old%20females%202014.pdf.

[11] Centre for Disease Control and Prevention. Teen vaccination coverage. [accessed 10.02.16]. http://www.cdc.gov/vaccines/who/teens/vaccination-coverage.html.

[12] Gerend M.A., Shepherd J.E. (2012). Predicting human papillomavirus vaccine uptake in young adult women: comparing the health belief model and theory of planned behavior. Ann Behav Med.

[13] Tickner S., Leman P.J., Woodcock A. (2007). ‘It's just the normal thing to do’: exploring parental decision-making about the ‘five-in-one’ vaccine. Vaccine.

[14] Guillaume L.R., Bath P.A. (2004). The impact of health scares on parents' information needs and preferred information sources: a case study of the MMR vaccine scare. Health Informatic J.

[15] Henderson L., Clements A., Damery S. (2011). ‘A false sense of security’? Understanding the role of the HPV vaccine on future cervical screening behaviour: a qualitative study of UK parents and girls of vaccination age. J Med Screen.

[16] Marlow L.A., Wardle J., Forster A.S. (2009). Ethnic differences in human papillomavirus awareness and vaccine acceptability. J Epidemiol Community Health.

[17] Hilton S., Hunt K., Petticrew M. (2007). Gaps in parental understandings and experiences of vaccine-preventable diseases: a qualitative study. Child Care Health Dev.

[18] Fontenot H.B., Domush V., Zimet G.D. (2015). Parental attitudes and beliefs regarding the nine-valent human papillomavirus vaccine. J Adolesc Health.

[19] Gordon D., Waller J., Marlow L.A. (2011). Attitudes to HPV vaccination among mothers in the British Jewish community: reasons for accepting or declining the vaccine. Vaccine.

[20] Stillo M., Carrillo Santisteve P., Lopalco P.L. (2015). Safety of human papillomavirus vaccines: a review. Expert Opin Drug Saf.

[21] Bowcott O. (2009). Girl who died after cervical cancer injection had tumour in her chest.

[22] Public Health England (2014). Human Papillomavirus (HPV) Vaccine coverage in England, 2008/09 to 2013/14.

[23] Morimoto A., Ueda Y., Egawa-Takata T. (2015). Effect on HPV vaccination in Japan resulting from news report of adverse events and suspension of governmental recommendation for HPV vaccination. Int J Clin Oncol.

[24] Freed G.L., Clark S.J., Butchart A.T. (2010). Parental vaccine safety concerns in 2009. Pediatrics.

[25] Van Damme P., Bonanni P., Bosch F.X. (2016). Use of the nonavalent HPV vaccine in individuals previously fully or partially vaccinated with bivalent or quadrivalent HPV vaccines. Vaccine.

[26] Ajzen I. (1985). From intentions to actions: the theory of planned behaviour.

[27] Rosenstock I.M., Strecher V.J., Becker M.H. (1988). Social learning theory and the Health Belief Model. Health Educ Q.

[28] Fu L.Y., Bonhomme L.A., Cooper S.C. (2014). Educational interventions to increase HPV vaccination acceptance: a systematic review. Vaccine.

[29] Niccolai L.M., Hansen C.E. (2015). Practice- and community-based interventions to increase human papillomavirus vaccine coverage: a systematic review. JAMA Pediatr.

[30] Opel D.J., Mangione-Smith R., Robinson J.D. (2015). The influence of provider communication behaviors on parental vaccine acceptance and visit experience. Am J Public Health.

[31] Skinner S.R., Davies C., Cooper S. (2015). HPV.edu study protocol: a cluster randomised controlled evaluation of education, decisional support and logistical strategies in school-based human papillomavirus (HPV) vaccination of adolescents. BMC Public Health.

